# *CERES*: a cryo-EM re-refinement system for continuous improvement of deposited models

**DOI:** 10.1107/S2059798320015879

**Published:** 2021-01-01

**Authors:** Dorothee Liebschner, Pavel V. Afonine, Nigel W. Moriarty, Billy K. Poon, Vincent B. Chen, Paul D. Adams

**Affiliations:** a Molecular Biosciences and Integrated Bioimaging, Lawrence Berkeley National Laboratory, 1 Cyclotron Road, Berkeley, CA 94720, USA; b Department of Biochemistry, Duke University, Durham, NC 27710, USA; c Department of Bioengineering, University of California Berkeley, Berkeley, CA 94720, USA

**Keywords:** cryo-EM, *Phenix*, re-refinement, *CERES*, scientific web pages

## Abstract

The field of electron cryomicroscopy (cryo-EM) has advanced quickly in recent years as the result of numerous technological and methodological developments. This has led to an increase in the number of atomic structures determined using this method. Recently, several tools for the analysis of cryo-EM data and models have been developed within the *Phenix* software package, such as *phenix.real_space_refine* for the refinement of atomic models against real-space maps. Also, new validation metrics have been developed for low-resolution cryo-EM models. To understand the quality of deposited cryo-EM structures and how they might be improved, models deposited in the Protein Data Bank that have map resolutions of better than 5 Å were automatically re-refined using current versions of *Phenix* tools. The results are available on a publicly accessible web page (https://cci.lbl.gov/ceres). The implementation of a *Cryo-EM Re-refinement System* (*CERES*) for the improvement of models deposited in the wwPDB, and the results of the re-refinements, are described. Based on these results, contents are proposed for a ‘cryo-EM Table 1’, which summarizes experimental details and validation metrics in a similar way to ‘Table 1’ in crystallography. The consistent use of robust metrics for the evaluation of cryo-EM models and data should accompany every structure deposition and be reported in scientific publications.

## Abstract 2

Atomic models derived from cryo-EM data with map resolutions of better than 5 Å were automatically re-refined. The results of the computations are publicly available on a web page.

## 1. Introduction   

**Figure 1:**
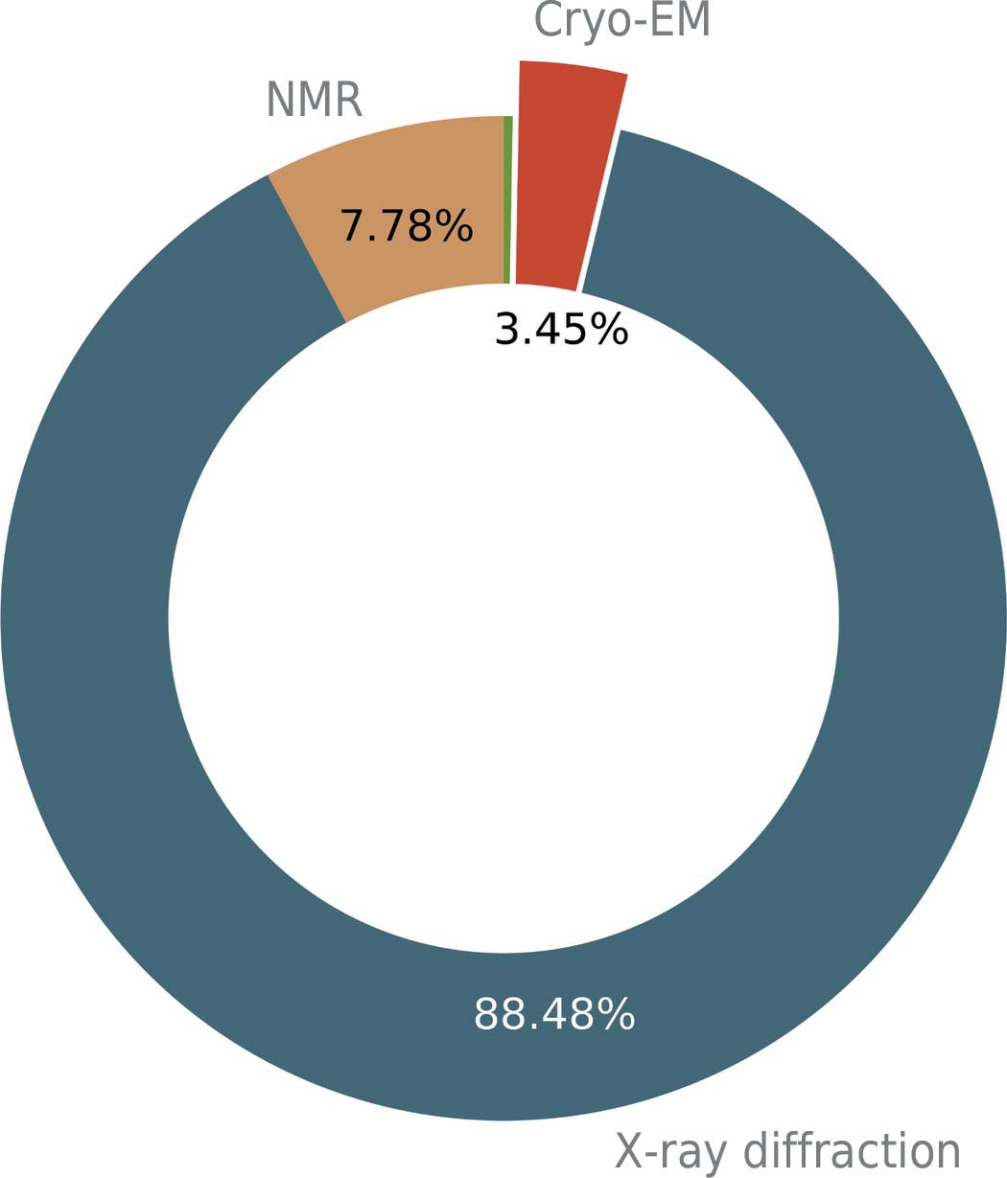
Cryo-EM is the third most used method for macromolecular structure determination after X-ray crystallography and nuclear magnetic resonance (NMR). Statistics were retrieved from the PDB on 10 May 2020. The total number of models in the PDB was 169 613. The green slice without a label represents the 485 models (0.3%) that were determined using other methods, such as neutron diffraction and electron diffraction.

**Figure 2:**
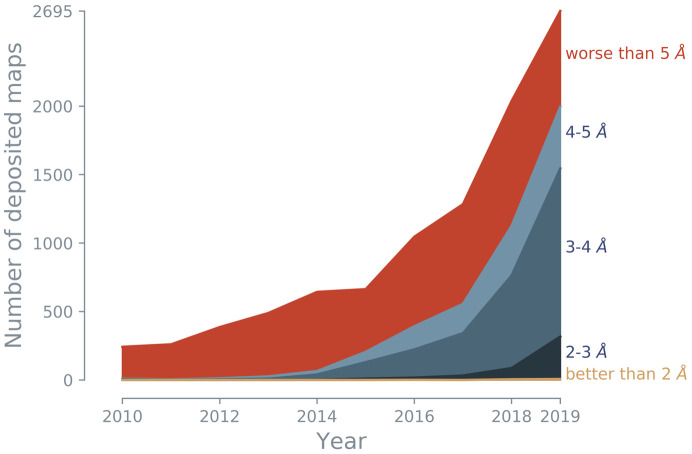
The number of cryo-EM maps has grown rapidly since 2013. In particular, the fraction of cryo-EM maps with resolutions of better than 5 Å (blue shaded areas) increased significantly. (Note that this figure shows the total number of maps, *i.e.* the maps do not necessarily have an associated model in the PDB).

Cryo-EM is an experimental technique that in the past has commonly been used to investigate large protein complexes, filaments and viruses. While the method was often limited to low resolution (5–9 Å), technological advances, such as the development of direct electron detectors (Faruqi *et al.*, 2003 ▸; Milazzo *et al.*, 2005 ▸; Deptuch *et al.*, 2007 ▸; Li *et al.*, 2013 ▸) and improvements in image processing (Campbell *et al.*, 2012 ▸; Scheres, 2012 ▸; Bai *et al.*, 2015 ▸), have led to an exponential increase in the number of cryo-EM models deposited in the Protein Data Bank (PDB; Berman *et al.*, 2000 ▸; wwPDB Consortium, 2019 ▸). As a consequence, cryo-EM is now the third principal method for macromolecular structure determination (Fig. 1 ▸), representing 3.1% of deposited models in the PDB. While this is currently behind X-ray crystallography (88.8%) and nuclear magnetic resonance (NMR; 7.9%), some researchers project that deposition numbers will reach those of crystallography in only five years (Hand, 2020 ▸). The advances in cryo-EM technology have led to greatly improved resolutions of the deposited 3D reconstructions (Fig. 2 ▸). Low-resolution cryo-EM density maps can be used to dock models from X-ray crystallography or NMR, but density maps of 5 Å resolution or better can be used to solve structures *de novo* and to refine atomic models, similar to X-ray crystallography. More recently, the majority of deposited maps have resolutions of better than 5 Å (Figs. 2 ▸ and 3 ▸), with 1.15 Å currently being the highest (EMDB entry EMD-11668, PDB entry 7a6a; K. M. Yip, N Fischer, A. Chari & H. Stark, unpublished work).

Cryo-EM 3D reconstructions are similar to the density maps derived from X-ray diffraction experiments. Hence, the tools developed for the building and refinement of crystallo­graphic models can be readily modified for application to cryo-EM. Intensities from a diffraction experiment do not contain phase information,^1^ which is necessary to compute (X-ray) density maps, and refinement is therefore typically performed in reciprocal space. In contrast, cryo-EM reconstructions are real-space maps, making refinement in real space a natural choice (Afonine, Poon *et al.*, 2018 ▸).

Best practices for the validation of cryo-EM maps and models have not yet been established. The validation criteria developed for crystallographic models can readily be applied to cryo-EM, as macromolecular stereochemistry obeys the same principles regardless of the experimental technique. However, data quality and model-to-data fit need to be formulated specifically for cryo-EM data. The process of finding the best validation parameters is therefore still ongoing, although progress has been made (Barad *et al.*, 2015 ▸; Afonine, Klaholz *et al.*, 2018 ▸; Williams *et al.*, 2018 ▸; Richardson, Williams, Videau *et al.*, 2018 ▸; Sobolev *et al.*, 2020 ▸). In particular, the EMDataResource team (https://challenges.emdataresource.org/) works with the cryo-EM community to establish validation methods for the structure-determination process. As suggested by the EM Validation Task Force (Henderson *et al.*, 2012 ▸), the team hosts benchmark challenges to stimulate community discussion about validation procedures. The most recent challenge is about model validation (Lawson *et al.*, 2020 ▸).

The *Phenix* software (Liebschner *et al.*, 2019 ▸) offers a series of programs that focus on the analysis of cryo-EM maps and models. For example, *phenix.real_space_refine* refines atomic models against real-space maps (Afonine, Poon *et al.*, 2018 ▸), and the ‘Comprehensive validation’ tool performs model validation using established geometry criteria from X-ray crystallography, based on *MolProbity* (Davis *et al.*, 2007 ▸; Chen *et al.*, 2010 ▸; Richardson, Williams, Hintze *et al.*, 2018 ▸; Williams *et al.*, 2018 ▸), and calculates cryo-EM-specific data and model-versus-data quality indicators (Afonine, Klaholz *et al.*, 2018 ▸). To improve a cryo-EM map, the following tools can be used: automatic map sharpening (*phenix.auto_sharpen*; Terwilliger *et al.*, 2018 ▸), density modification (*phenix.resolve_cryo_em*; Terwilliger, Ludtke *et al.*, 2020 ▸; Terwilliger, Sobolev *et al.*, 2020 ▸) and *phenix.combine_focused_maps* (recombination of the best parts of several maps). An initial atomic model representing a cryo-EM map can be obtained with *phenix.dock_in_map* and *phenix.map_to_model* (Terwilliger, Adams *et al.*, 2020 ▸).

In light of these very recent advances in refinement and validation, it is worthwhile revisiting previously deposited cryo-EM structures. Much can be gained by re-refining each entry and assessing model and data quality. The cryo-EM field is evolving at such a rapid pace that recently deposited models were obtained (i) using the software and methods available at the time but which have since changed markedly and (ii) when no community-wide accepted consensus about validation was established. Therefore, re-refining cryo-EM structures with current methods represents an opportunity to obtain a snapshot of data and model quality based on consistent refinement algorithms and validation criteria. Efforts to re-refine crystallographic models against diffraction data with the *PDB-REDO* procedure (Joosten, Salzemann *et al.*, 2009 ▸; Joosten *et al.*, 2014 ▸) have proved to be a great success. Brown *et al.* (2015 ▸) showed that cryo-EM models that were determined from maps with a resolution of 4 Å or better could be improved after refinement. Another benefit of re-refining a large number of models is that new computational methods and procedures can be tested for success, stability and validity. In this way, continuous improvement of the software and of the methods can be ensured.

In this work, we re-refined cryo-EM models with maps with a resolution of 5 Å or better. The results are available on a publicly accessible web page. Each processed map–model pair has an individual page that displays map, model and map-to-model validation statistics. A molecular viewer is incorporated, allowing easy visualization of maps and models along with identification of obvious issues. All re-refinement results can be also accessed in a table, letting the user browse for models with particular properties (for example, map resolution, model statistics or number of residues). We also suggest a set of metrics for model, data and model-versus-data quality (‘cryo-EM Table 1’) that should accompany every structural publication.

## 2. Materials and methods   

### 2.1. Re-refinement procedure   

**Figure 4:**
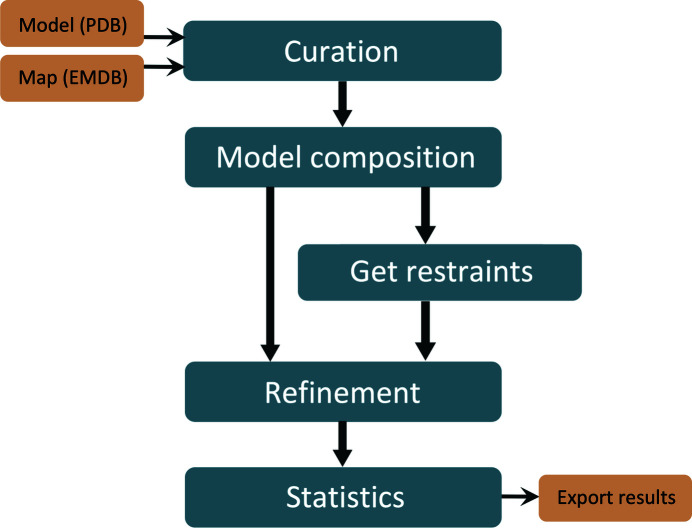
Steps in the re-refinement procedure. The process is divided into tasks that are executed sequentially or on demand.

The automated re-refinement procedure is divided into different tasks; a flow chart of the steps is shown in Fig. 4 ▸. All steps are described in detail in the following subsections. In summary: firstly, the maps and models are downloaded from the respective repositories and, if necessary, curated. Subsequently, the model composition is analyzed and chemical restraints for nonstandard compounds are created. The structure is then refined against the cryo-EM map using *phenix.real_space_refine* followed by examination of the statistics for the initial and the refined model. Finally, the results are exported and stored in a database. The script is based on code from the *Computational Crystallography Toolbox* (*cctbx*; Grosse-Kunstleve *et al.*, 2002 ▸) and the *Phenix* software (Liebschner *et al.*, 2019 ▸). Scripts are available in the RErefine GitHub repository (https://github.com/pafonine/CryoEMRErefine).

#### 2.1.1. Obtaining maps and models   

For structures determined by cryo-EM, the models and the maps are stored in different repositories, namely the PDB and the Electron Microscopy Data Bank (EMDB; Lawson *et al.*, 2011 ▸). Corresponding map–model pairs (model file, map files and half-maps if available) are downloaded to local storage for further processing. We note that one map can be associated with several models or one model can be associated with several maps.

#### 2.1.2. File preparation and curation   

Models available in mmCIF format were converted to PDB format.^2^ If only the unique unit of a biological assembly was present in the model, the assembly was generated with the symmetry operators indicated in the header. The model and map files may each contain one or several values for the resolution of the map. In the majority of cases the values are the same, but if they were different then the map–model pair was not considered for re-refinement. The resolution that is indicated in both the model and map files is the ‘consensus resolution’. Maps and/or model(s) were not considered for further processing if one of the following conditions was met.

(i) Maps without a corresponding model in the PDB (not present or not yet released).
(ii) Resolution mismatch within files (no ‘consensus resolution’).
(iii) EMDB entries where maps and half-maps have different gridding.
(iv) Mismatch of reconstruction box dimensions between files or nonsensical values for dimensions.
(v) Failure to process maps or models with *cctbx* tools.
(vi) Ensemble models (multi-model files).
(vii) More than 25% of the residues of a model consist of a single atom (for example, C^α^- or P-atom models).
(viii) A consensus resolution worse than 5 Å.

#### 2.1.3. Model composition and ligand restraints   

Model information, such as the number of ligands, residues and chains, the atomic displacement parameters (ADPs) and the occupancy statistics, were extracted from the PDB files created in the previous step. When a model contained ligands, it was sometimes necessary to supply geometry restraints for refinement. In these cases, ligand restraints were obtained with *phenix.ready_set*, which uses *phenix.elbow* (Moriarty *et al.*, 2009 ▸).

#### 2.1.4. Refinement   

Refinements were performed with *phenix.real_space_refine*. The number of refinement macrocycles was set to ten; electron scattering factors were used. Symmetry-related chains were detected automatically using the *phenix.simple_ncs_from_pdb* tool. If plausible copies are found, the operators relating them are derived and used to check whether the map was symmetrized (*i.e.* molecular symmetry was imposed during the reconstruction) using *phenix.map_symmetry* (Liebschner *et al.*, 2019 ▸). Symmetry constraints are applied if the map was symmetrized or the resolution was worse than 4.5 Å. In addition to standard stereochemical and nonbonded restraints, we applied secondary-structure restraints (Headd *et al.*, 2012 ▸) for protein and nucleic acid residues, Conformation-Dependent Library (CDL) restraints (Moriarty *et al.*, 2016 ▸), C^β^-deviation restraints, rotamer restraints and Ramachandran plot restraints (Headd *et al.*, 2012 ▸). The resolution limit for refinement was set to the consensus resolution. The resolution does not affect the refinement results, but is used to calculate and report the map–model correlation coefficient (Afonine, Poon *et al.*, 2018 ▸).

#### 2.1.5. Statistics and plots   

Validation of both the input structures and refined structures was performed. This allowed validation of the refinement step as well as a comparison of the properties of the initial and refined structures, model parameters, data quality and the model-to-data fit. For validation, the same resolution cutoff as for refinement was applied. Most of the metrics are standard and are well documented in the crystallographic literature (Hooft *et al.*, 1997 ▸; Chen *et al.*, 2010 ▸; Read *et al.*, 2011 ▸; Gore *et al.*, 2017 ▸; Wlodawer, 2017 ▸; Williams *et al.*, 2018 ▸). Novel metrics that are specific to cryo-EM are summarized in Section 3.2. Plots were generated with *Matplotlib*, a Python 2D plotting library (Hunter, 2007 ▸).

#### 2.1.6. Monthly computations   

For smaller models (PDB file size <20 MB), the models are re-refined once a month. By default, the search function and the table show the results from the previous month (as the results of the current month are typically still being processed). Older results can be accessed as well, which allows comparison of the runs from different months. As larger models require significantly longer processing times, they are processed every six months.

### 2.2. Design of the website   

The results of the re-refinements are stored in a database (PostgreSQL; https://www.postgresql.org). The webpage was created with Django (https://www.djangoproject.com), a web framework following the model–view–controller architectural pattern. Several common JavaScript and CSS libraries are used to create responsive content and tables: Bootstrap (https://getbootstrap.com), JQuery (https://jquery.com), Djangotables 2 (https://django-tables2.readthedocs.io/en/latest/) and Maphighlight (https://github.com/kemayo/maphilight). The NGL viewer (Rose & Hildebrand, 2015 ▸; Rose *et al.*, 2018 ▸), a library for molecular visualization, is embedded into each page of individual results, allowing visualization of the maps and models (initial and refined).

## 3. Results and discussion   

As of June 2020, the EMDB contains about 11 000 maps. Approximately 4500 of these have corresponding models in the PDB. Among the maps with a corresponding model in the PDB, ∼3300 have a resolution of 5 Å or better, with ∼350 of them not passing the map and model curation step. As of the time of preparation of this manuscript, ∼2750 map–model pairs successfully passed at least one step of the re-refinement procedure^3^ and the results are displayed on the *CERES* website https://cci.lbl.gov/ceres.

### 3.1. Filtered models   

To be able to automatically re-refine models against maps, it is necessary to ensure that the maps and models pass some basic consistency checks. For example, of the 3308 map–model pairs (in local storage on 26 June 2020), 370 (more than 10%) did not pass the curation. The majority of failures are due to inconsistent resolution information in the files (263). Other failures are caused by inconsistent gridding in maps and half-maps (56), bad symmetry (or box) information (18) and the model consisting of more than 25% single-atom residues (17). The minority of failures are due to processing issues with *Phenix* tools (16). An example of inconsistent resolutions is PDB entry 6sfw [10162]:^4^ the resolution limit indicated in the map file is 4 Å, while that in the model (mmCIF format) file is 6 Å. A comment in the mmCIF file informs that although the overall resolution of the map is 4 Å, the region in which the molecule was modeled has a resolution of only 6 Å. Unfortunately, it is not practical to automatically screen comments that can explain the mismatch, leading to the removal of the entry from the refinement list. Inconsistent gridding involving half-map files is not necessarily a problem for refinement, but such obvious disagreements are often indicative of other issues. Therefore, these instances are ignored in further processing. An example of inconsistent box information is PDB entry 6udk [20740]. A cryo-EM map is expressed as a three-dimensional array of density values inside a ‘map box’. For PDB entry 6udk, the cell indicated in the map file is a cube of length 291.20 Å. However, the cell lengths indicated in the model file are 1.0 Å. In such cases, the coordinates in the model file might actually correspond to a model that has been placed in a box with the same lengths as indicated in the map file. However, another possible scenario could be that the deposited map is in a box that is much larger than the molecule, while the molecule coordinates are expressed in a smaller box. In other instances, such as PDB entry 6hug [0275], the model file has no symmetry information at all.

The above examples of maps and models that failed the curation step underscore the necessity to store information consistently and clearly in the map and model metadata. Otherwise, errors in bookkeeping or ambiguous metadata can lead to erroneous results in automated data-mining efforts, such as structure-guided drug design (Dauter *et al.*, 2014 ▸). Data-mining projects in crystallography from the Electron Density Server or *PDB-REDO* faced comparable issues (Kleywegt *et al.*, 2004 ▸; Joosten, Salzemann *et al.*, 2009 ▸; Joosten, Womack *et al.*, 2009 ▸). Therefore, the information in the databases should be well curated and unambiguous so that they are usable by experts and non-experts alike.

### 3.2. Cryo-EM Table 1   

Each re-refinement result is summarized in ‘Table 1’, which represents the most important metrics of overall model and data quality and model-versus-data fit, similar to ‘Table 1’ used in crystallography. While some quality indicators are identical for both, some are specific to cryo-EM. We recommend that new reports of cryo-EM structural studies include a cryo-EM Table 1, as it represents an expanded and amended version of the established Table 1 for crystallography. In the following, we briefly describe each indicator. This is not an exhaustive list, and newly developed metrics may eventually be added. The use of robust quality metrics by cryo-EM practitioners will enhance best practices of model building and refinement and will help in checking whether the model that is being built into the map is as correct as it could be.

#### 3.2.1. Model-quality indicators  

*Clashscore*. The clashscore represents the number of severe atomic clashes, which are pairs of atom van der Waals spheres overlapping by more than 0.4 Å per 1000 atoms (Word *et al.*, 1999 ▸; Williams *et al.*, 2018 ▸). In general, a higher quality structure will have fewer clashes and therefore a lower clashscore. Clashes are a particular issue in low-resolution maps (worse than 3 Å) and are a useful diagnostic for local misfitting. For example, as the density of side chains is often missing or incomplete at low resolution, this may cause side chains to adopt conformations that produce steric overlaps.
*Deviations from geometry target values*. The r.m.s.d. values show whether the refined model deviates from the dictionary values that are used for geometry restraints in refinement. At low resolution (worse than 3 Å), it is expected that the deviations are relatively small, as the atomic coordinates are not accurate enough to justify outliers. An even more satisfactory metric to assess deviations from ideal geometry is the r.m.s. *Z*-score (RMSZ). The *Z*-scores are obtained by calculating the difference between the observed and ideal target value, divided by the standard deviations of the target. RMSZ scores are expected to lie between 0 and 1.
*C^β^ deviations*. This metric reflects the number of instances where the distance between the observed location of the C^β^ atom and its ideal position (which can be inferred from the position of the other main-chain atoms) exceeds 0.25 Å. The distance represents distortions in the bond geometry around the C^α^ atom. For example, if the backbone and/or side chains are misfitted, the C^β^ atom may be moved far from the ideal position to compensate.
*Rotamer outliers*. Amino-acid side chains adopt certain sets of torsion angles. Some combinations are preferred, while others are not possible owing to steric clashes and other atomic interactions. The vast majority of side chains in a structure should be in favored rotamer states unless the map very clearly supports a rare or outlier conformation. Fitting side chains into cryo-EM maps can be challenging, as their density is often missing or incomplete at low resolution (worse than 3 Å). Therefore, few rotamer outliers are expected in cryo-EM models.
*Ramachandran outliers/favored/allowed*. As only certain backbone (Ramachandran) dihedral angle combinations are possible due to steric constraints, they are used as a validation measure for model geometry. The two-dimensional graph of the backbone angles is divided into ‘favored’, ‘allowed’ and ‘outlier’ regions. An accurate and well refined structure will approach having more than 98% of residues in the favored regions, with less than 0.2% outliers, although it may be difficult to obtain these statistics at lower resolutions.
*Rama-*Z* score*. The Ramachandran *Z*-score (Rama-*Z* score) characterizes the shape of the backbone angle distribution in the Ramachandran plot (Hooft *et al.*, 1997 ▸). Indeed, even if a refined model has satisfying Ramachandran statistics in terms of the fractions of residues belonging to favored/allowed/outlier regions, the distribution of backbone dihedrals can be improbable (Sobolev *et al.*, 2020 ▸). A normal backbone protein backbone geometry results in Rama-*Z* values between −2 and 2. A less likely yet possible distribution has absolute Rama-*Z* values between 2 and 3. A Rama-*Z* score with an absolute value above 3 corresponds to an improbable Ramachandran distribution.
*Minimum nonbonded distance*. This number represents the shortest distance between two atoms that are not covalently bonded. If the number is small, then it means that the two atoms may be clashing.
*CaBLAM outliers*. The *C^α^-Based Low-resolution Annotation Method* (*CaBLAM*) evaluates main-chain geometry (Williams *et al.*, 2018 ▸) and is particularly useful for diagnosing backbone conformational errors in lower resolution structures. Good structures are expected to have less than 1% *CaBLAM* outliers; a model with more than 5% outliers is problematic.

#### 3.2.2. Data-quality indicators  

**d*_FSC_*. The resolution obtained from the correlation of Fourier map coefficients between two half-maps, binned in resolution shells. It represents the maximum spatial frequency at which the information content can be considered to be reliable (Rosenthal & Henderson, 2003 ▸).
**d*_model_*. The resolution of the model-calculated map that maximizes its similarity to the experimental map (Afonine, Klaholz *et al.*, 2018 ▸).
*d_99_*. The resolution estimate related to map details. The value is obtained by gradually removing the highest resolution Fourier map coefficients. *d*_99_ is the resolution at which a map calculated from the reduced set of coefficients starts to differ from the original map (Afonine, Klaholz *et al.*, 2018 ▸).
*Consensus resolution*. In addition to the values above, cryo-EM Table 1 on the website reports the consensus resolution, which is derived from deposited files.

#### 3.2.3. Model-versus-data fit  

*CC_box_, CC_mask_*. The model–map correlation coefficient (CC) reflects how well a model fits to a map. CC_box_ and CC_mask_ use the entire map and the map values in an envelope around the molecule, respectively (Afonine, Klaholz *et al.*, 2018 ▸).
*EMRinger score*. A score reflecting the correctness of the main chain by using the side-chain information content of the maps (Barad *et al.*, 2015 ▸). The score is calculated for maps with resolutions better than 4 Å. At worse resolution, it is not expected to provide meaningful density values for side chains. An EMRinger score below 1.0 is problematic; while the score is resolution-dependent, it can be generally assumed that the higher the score, the better the correctness of the main chain.

### 3.3. Results from re-refinement   

Of the September 2020 data set, 2535 map–model pairs successfully passed through all steps of the re-refinement procedure. In the following, some of the quality indicators in cryo-EM Table 1 are discussed and compared for the initial and the re-refined structures.

We do not discuss the following indicators in detail. The geometry-restraints r.m.s.d. values are expected to be small in the resolution ranges typical for cryo-EM (although this may change in the future, with more and more maps determined at resolutions of 2 Å and better). Therefore, a decrease or increase does not necessarily mean that the structure is better or worse. We also do not show results for EMRinger score and *d*_99_ for the sake of brevity.

#### 3.3.1. Map-to-model fit   

**Figure 5:**
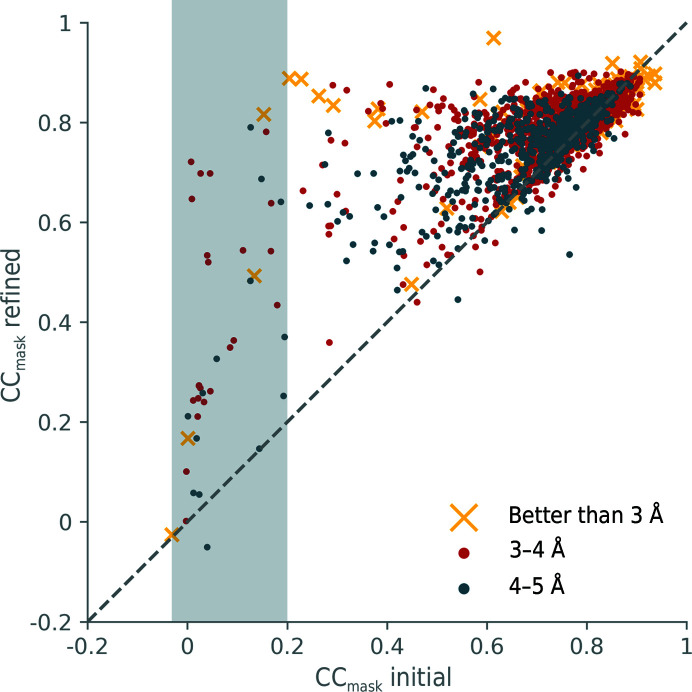
Map–model correlation CC_mask_ before (*x* axis) and after (*y* axis) refinement. The gray shaded area highlights models with a low initial CC_mask_ of <0.2. Different colors represent the consensus resolution of the map (see legend).

**Figure 6:**
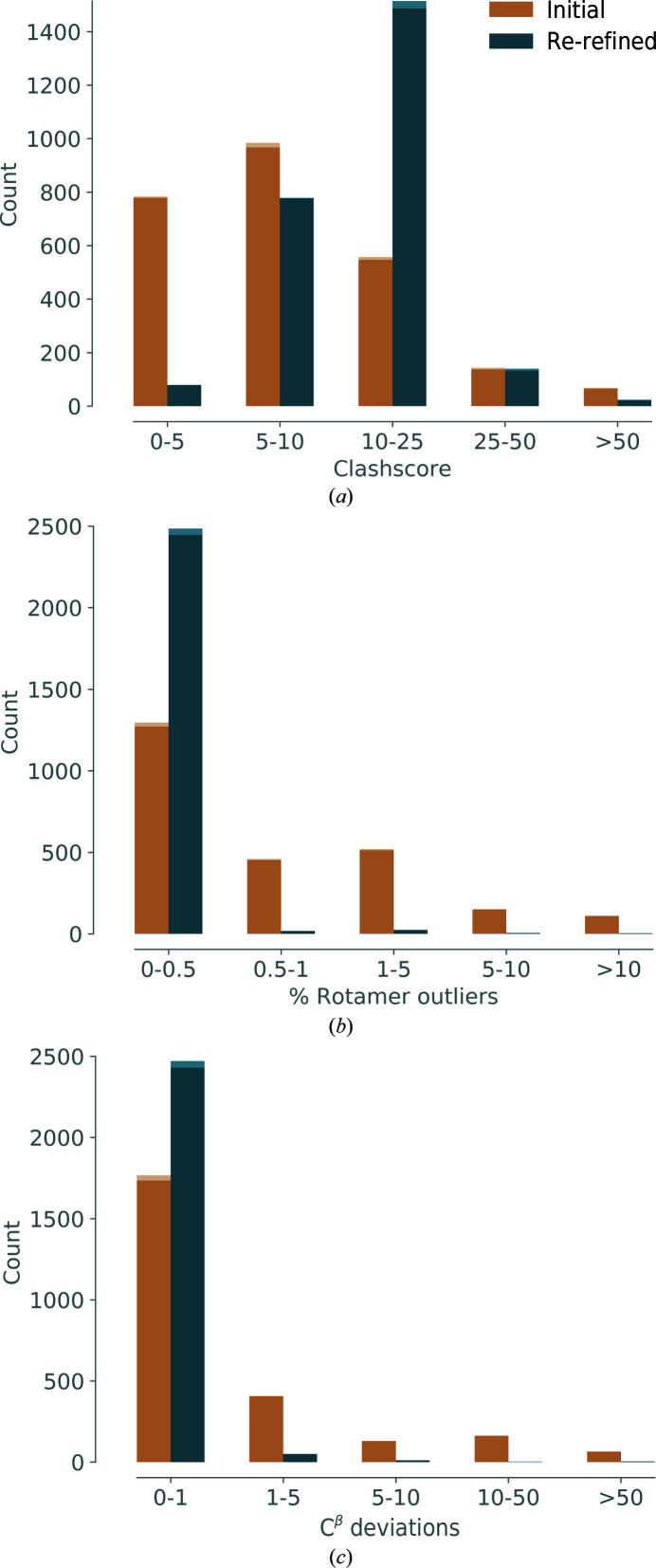
Statistics of models before refinement (yellow bars) and after refinement (blue bars): (*a*) clashscore, (*b*) % rotamer outliers, (*c*) C^β^ deviations.

In general, the map–model correlation coefficient (CC_mask_) reflects how well a model fits to a map. For a large majority of the re-refined models, the correlation coefficient CC_mask_ improves after real-space refinement (Fig. 5 ▸). When the initial CC_mask_ is high (>0.8), the improvement is relatively small. For initial CC_mask_ values between 0.4 and 0.8, the improvement is usually more substantial. We also observe an improvement for models that have low initial correlation coefficients (0–0.4). While this may reflect a genuine improvement, it can also be indicative of problems with the starting model and map. We note that the correlation coefficient should not be viewed as the single quality indicator of map-to-model fit, as there are scenarios in which it can be misleading. For example, if the CC is very low for one chain of a multi-chain model it decreases the overall correlation coefficient. Furthermore, a model with a good map–model correlation can have bad model-quality indicators. Nevertheless, the CC can be useful to flag serious problems. If the initial CC_mask_ is very low, it indicates that the model does not fit well to the map. This may occur if the deposited model does not superpose on the map, for example when it is shifted or rotated (or both) with respect to the map. Among the structures that were re-refined, 41 models have initial correlation coefficients smaller than 0.2 (gray shaded area in Fig. 5 ▸). In Figs. 6 ▸, 7 ▸ and 8 ▸, which show histograms for model-quality indicators, models that have initial CC values below 0.2 are highlighted with a lighter color. In this way, it can be seen whether models that have a low initial model-to-map fit result in models with suboptimal geometry.

An example of a model with a low initial CC is PDB entry 6eu1 [3956]; the initial value for CC_mask_ is 0.01, with the model being slightly shifted with respect to the map. The re-refined model yields a CC_mask_ value of 0.72, with an average shift of more than 2 Å compared with the initial structure. While the map–model fit is visibly improved, the model geometry deteriorates: the clashscore increases from 6.5 to 17.7 and the percentage of residues in the favored Ramachandran region only marginally improves from 83.5% (which is already poor) to 83.7%. The default real-space refinement procedure for individual coordinates is designed to improve the local details of the model. Larger scale changes require the application of simulated annealing (Grosse-Kunstleve *et al.*, 2009 ▸) or morphing options (Terwilliger *et al.*, 2013 ▸). However, very large-scale movements such as significant shifts or rotations of entire molecules or chains are outside the radius of convergence of the real-space refinement procedures. Therefore, for maps and models that yield very poor correlations before refinement, a different strategy would be required. For example, the model could be first refined as a rigid body, or better still the model could be docked into the map with *phenix.dock_in_map*. These strategies will be explored in further versions of the re-refinement server.

While there is a slight tendency for higher resolution maps to yield a higher CC_mask_, manifested by slightly shifted distributions for the resolution ranges better than 3 Å, 3–4 Å and 4–5 Å, the trend is not obvious enough to postulate that higher resolution maps generally have better CC values. This is unlike X-ray crystallography, where *R* factors are somewhat correlated with resolution (Joosten, Salzemann *et al.*, 2009 ▸; Read & Kleywegt, 2009 ▸; Urzhumtsev *et al.*, 2009 ▸).

#### 3.3.2. Geometry  

**Figure 7:**
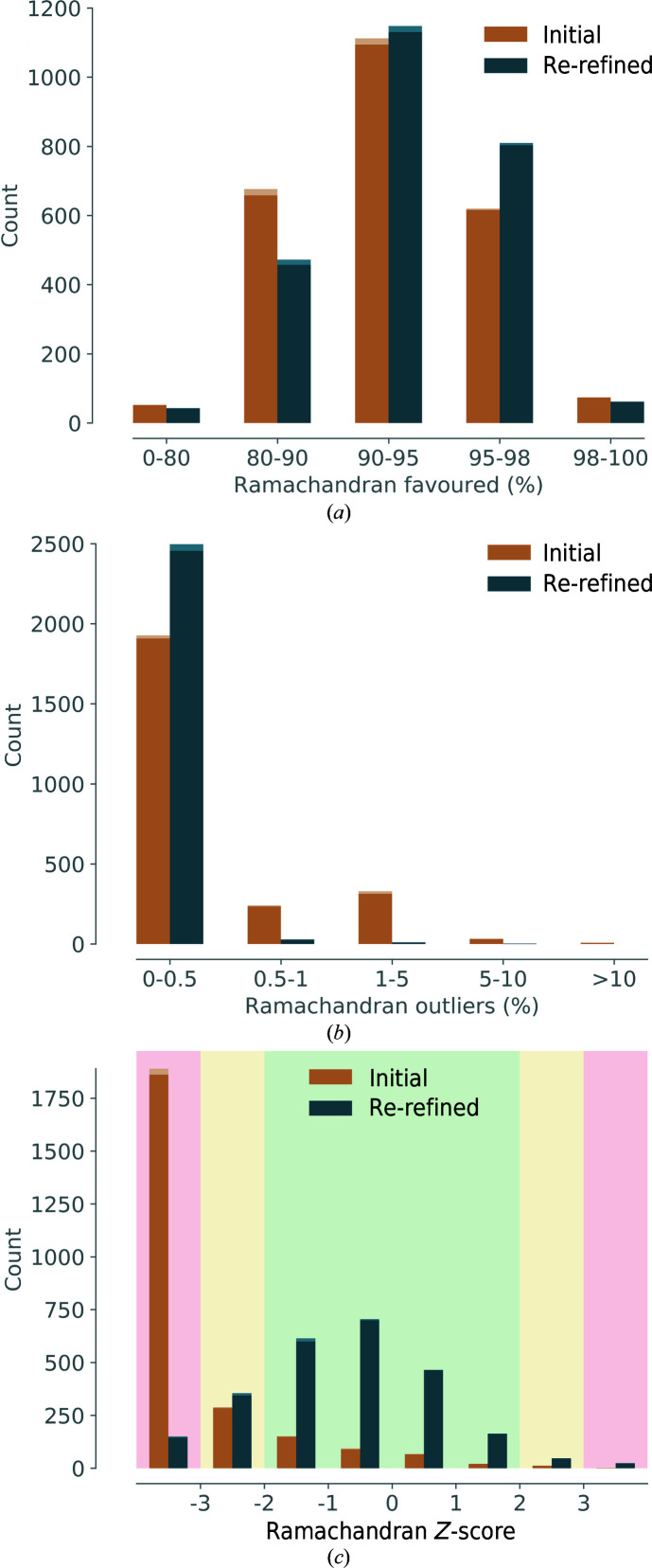
Statistics before and after refinement: (*a*) Ramachandran outliers, (*b*) Ramachandran favored, (*c*) Rama-*Z* score, with the background color indicating quality (green, good, yellow, suspicious, red, poor).

**Figure 8:**
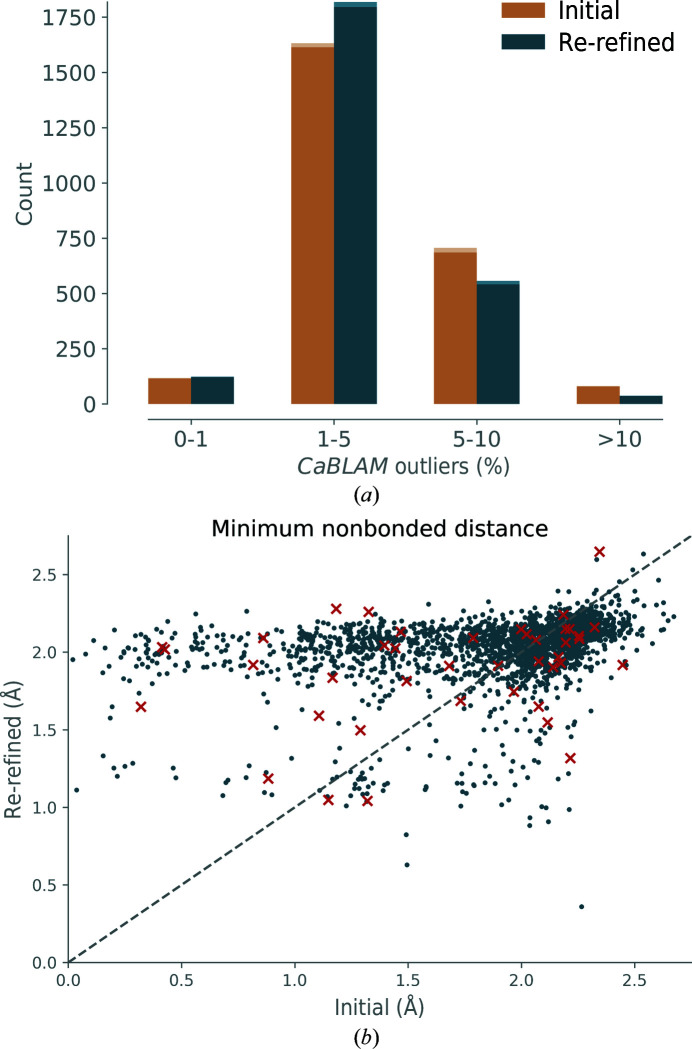
Statistics of models before and after refinement. (*a*) *CaBLAM* outliers. (*b*) Minimum nonbonded distance; red crosses, models with initial CC_mask_ ≤ 0.2.

*Clashscore*. Fig. 6 ▸(*a*) shows a histogram of clashscore values for the initial and re-refined models. We note that the clashscore increases for the re-refined models: the center of the distribution shifts from a clashscore of between 5 and 10 to values of between 10 and 25. There are several possible reasons for this change. Firstly, the initial clashscore values are most likely to be artificially low. Clashes between neighboring atoms are typically minimized by including a nonbonded restraint term in the geometry restraints used in refinement. If the weight of this nonbonded restraint is made artificially large, it will prevent clashing atoms, but at the same time it will not correctly reflect the interaction potential between non­bonded atoms. It is possible that some of the structures with very low clashscores were obtained with an inappropriate non­bonded weight. A second reason for the increasing clashscore could be the lack of H atoms in the model. About half of the atoms in a typical macromolecule are H atoms. However, although they represent a significant amount of matter in the sample, they are typically not included in models. Instead, the models contain almost exclusively the ‘heavier’ atoms N, C, O and S. While the clashscore is calculated from a model where H atoms have been added, the model is refined without H atoms. It is therefore possible that the nonbonded restraints in the *phenix.real_space_refine* procedure are not optimal in the absence of H atoms. A third reason is that clashes may have been minimized in the model at the expense of the other stereochemical parameters described below. After the refinement has made improvements across the whole structure, these other metrics are improved at the cost of a small increase in the number of clashes. We note that the number of models with unreasonably high clashscores (>50) decreases after re-refinements.
*Rotamers*. The percentage of rotamer outliers for the initial and re-refined structures is shown in Fig. 6 ▸(*b*). The rotamer outliers for the initial models can reach more than 10%. For the large majority of re-refined models, the percentage of rotamer outliers is 0–0.5%. Therefore, the side-chain conformations generally improve after refinement.
*C^β^ deviations*. A histogram of the number of C^β^ deviations is shown in Fig. 6 ▸(*c*). Most initial models have one or zero C^β^ deviations, but over 220 models have over ten. In contrast, the large majority of re-refined models have zero to one C^β^ deviations, and only very few reach more than ten. As the C^β^ deviations reflect distortions around the C^α^ atom, this means that the geometry of the re-refined models has generally improved.
*Ramachandran favored*. The histogram of favored Ramachandran angles is shown in Fig. 7 ▸(*a*). The center of the distribution is at 90–95% for the initial models. While the majority of re-refined models have the peak of the distribution at the same percentage, the number of residues with dihedral backbone angles in the 80–90% region and lower decreases. Therefore, the Ramachandran distribution for favored angles improved overall. There is still a non-negligible number of models that have a poor favored percentage (lower than 90%). It is possible that this is owing to the application of Ramachandran restraints. As the density in low-resolution maps lacks clear features to precisely locate backbone atoms in refinement, Ramachandran restraints are a possibility to maintain the expected Ramachandran distributions. Different potentials for the Ramachandran target functions exist (Oldfield, 2001 ▸; Emsley *et al.*, 2010 ▸; Headd *et al.*, 2012 ▸) and their implementation will benefit from further improvements. For example, the target in Oldfield-like Ramachandran restraints depends on the dihedral angles in the starting model. If these angles are incorrect and/or far from the ‘real’ values, the target can guide the backbone angles to suboptimal regions. It should be noted that the Ramachandran plot is not an independent validation metric if it was used as a source of restraints during refinement. However, it still reflects model quality, similar to the deviations from bond-length and angle target values, which are usually applied as restraints in refinement.
*Ramachandran outliers*. A histogram of the percentage of Ramachandran outliers is shown in Fig. 7 ▸(*b*). A significant number of initial models have Ramachandran outliers between 0.5 and 5%, which is far more than is considered to be an acceptable value for well refined structures. In contrast, the large majority of re-refined models have a percentage of outliers in the lowest range (0–0.5%). The percentage of Ramachandran outliers therefore generally improves after refinement.
*Rama-*Z* score*. The Rama-*Z* score for the initial and re-refined models is shown in Fig. 7 ▸(*c*). The majority of initial structures have absolute Rama-*Z* values above 3 (red shading), indicating that their Ramachandran angle distribution is problematic. After re-refinements, many structures have an acceptable (2 < |Rama-*Z*| < 3, yellow shading) or even high-quality (|Rama-*Z*| < 2, green shading) metric.
**CaBLAM* outliers*. A histogram of *CaBLAM* outliers is shown in Fig. 8 ▸(*a*). Most initial and re-refined models have 1–5% *CaBLAM* outliers, which is more than the percentage considered high quality for well refined structures (up to 1%). Moreover, a significant amount of initial and re-refined models have more than 5% of outliers, which is considered problematic. *CaBLAM* outliers can flag suspicious main-chain conformations that are in the wrong region of the Ramachandran plot even when adopting favored or allowed di­hedral angles. The histogram of the re-refined models has a similar distribution to that of the initial models, but there are fewer models with 5–10% and >10% outliers.
*Minimum nonbonded distance*. A scatter plot of the minimum nonbonded distance is shown in Fig. 8 ▸(*b*). Points located above the diagonal line represent refinements where the minimum distance increases. This is especially desired if the minimum distance is small in the initial model. For example, the minimum distance is smaller or equal to 1 Å in 145 initial models, while only eight re-refined models have such short distances. It is unlikely that all of these short distances represent genuine noncovalent interactions, so this increase represents a clear improvement. However, there are some minimum distances that are between 1 and 2 Å in the initial model and that are reduced in the re-refined model (points located below the diagonal and between 1 and 2 Å on the *x* axis). It is likely that these cases are related to the refinements that lead to poor clashscores (Fig. 6 ▸ *a*).

#### 3.3.3. Data resolution   

The current standard for determining the resolution of cryo-EM reconstructions is the frequency-dependent comparison (Fourier shell correlation analysis; FSC) of half-maps. The nominal resolution *d*_FSC_ of a map is where the FSC between half-maps is about 0.143, which corresponds to an estimated correlation of 0.5 between the experimentally determined map and the (unknown) true map (Rosenthal & Henderson, 2003 ▸). We note that other cutoff values, such as 0.5, may also be used (Böttcher *et al.*, 1997 ▸; Frank, 2006 ▸). While the resolution does not affect the refinement results, it is used to calculate the map–model correlation coefficient. Furthermore, the resolution is used as a criterion for filtering out candidates to be re-refined, *i.e.* models with maps that have a resolution worse than 5 Å are not refined. If the consensus resolution obtained from file headers is better than 5 Å but the real resolution of the map is lower, then the real-space refinement procedure may not provide optimal results. It is therefore of interest to be able to filter out these cases.

**Figure 9:**
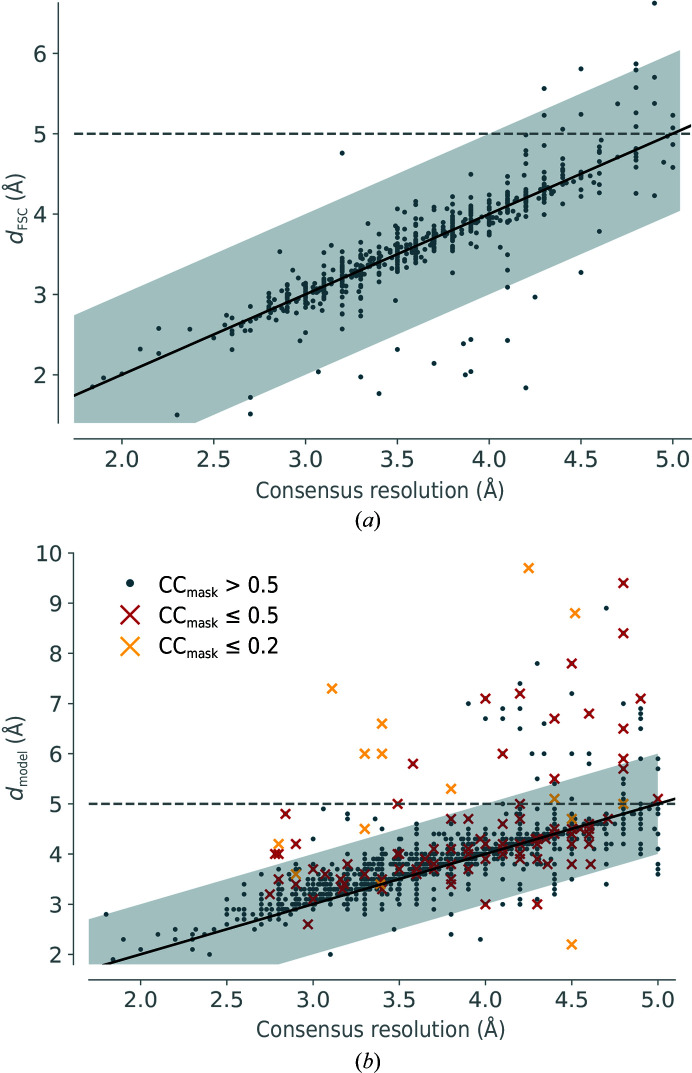
Resolution estimates compared. (*a*) *d*_FSC_ versus the consensus resolution. (*b*) *d*_model_ versus the consensus resolution. 22 models have *d*_model_ worse than 10 Å. They are not shown in order to maintain the focus of the graph on the axis diagonal.

A scatter plot of the recalculated *d*_FSC_ (from half-maps, if available) plotted against the consensus resolution is shown in Fig. 9 ▸(*a*). Unfortunately, not all EMDB depositions include half-maps, so *d*_FSC_ could not be recalculated for all maps that were used for re-refinements. Only 659 of the 2535 map–model pairs had half-maps (∼25%). For these maps, the *d*_FSC_ from half-maps is generally within 1 Å of the consensus resolution (gray shaded area around the diagonal). In some cases, *d*_FSC_ is significantly better than the consensus resolution. It is possible that in these instances the resolution was determined using a stricter cutoff than 0.143 for the FSC. In only 18 cases was *d*_FSC_ worse than 5 Å, with the worst *d*_FSC_ being 6.62 Å. It may be that there was some error in depositing the half-maps in this case. It therefore seems that the reported consensus resolution is generally quite close to the re-calculated *d*_FSC_ value. An alternative method to estimate the resolution for the other ∼75% of maps are *d*_model_ and *d*_99_. *d*_model_ is shown in Fig. 9 ▸(*b*). Generally, *d*_model_ is within 1 Å of the consensus resolution. There are significant outliers, which as expected occur for models that have a very low initial model-to-map correlation (CC_mask_ lower than 0.2, yellow crosses). For these cases, the resolution estimate *d*_model_ is likely to be flawed. For models with initial CC_mask_ values between 0.2 and 0.5, *d*_model_ is often close to the consensus resolution, but may differ significantly (red circles). The remaining cases where CC_mask_ > 0.5 and *d*_model_ is worse than 5 Å are candidates to be filtered out.

Intuitively, one expects *d*_model_ to be most reliable when the model fits the map best, *i.e.* if the re-refined model fits better to the map, then its *d*_model_ should be better than the initial value. However, it has been previously observed that this is not the case (Afonine *et al.*, 2018 ▸). It is possible that the *d*_model_ values do not follow this correlation owing to unusual atomic displacements or map peculiarities such as non-uniform resolution across the map volume.

For example, PDB entry 6i52 [4410] has a consensus resolution of 4.7 Å, while *d*_model_ is equal to 8.9 Å. The deposited map has a visibly lower resolution than 4.7 Å (https://cci.lbl.gov/ceres/goto_entry/6i52_4410/09_2020). In this case, it is possible that the consensus resolution corresponds to a map that has been processed in some way, such as sharpening. Future re-refinements will include filtering for maps that have a low *d*_model_ and at the same time an acceptable CC_mask_. This way, maps that are likely to have a lower resolution than reported can be excluded from re-refinement.

### 3.4. Examples   

The following section discusses two examples where the automatic re-refinement procedure led to models with better model and model-versus-data metrics. Each case exemplifies the features of *phenix.real_space_refine* at different resolution ranges.

#### 3.4.1. PDB entry 5k12, 1.8 Å resolution   

**Figure 10:**
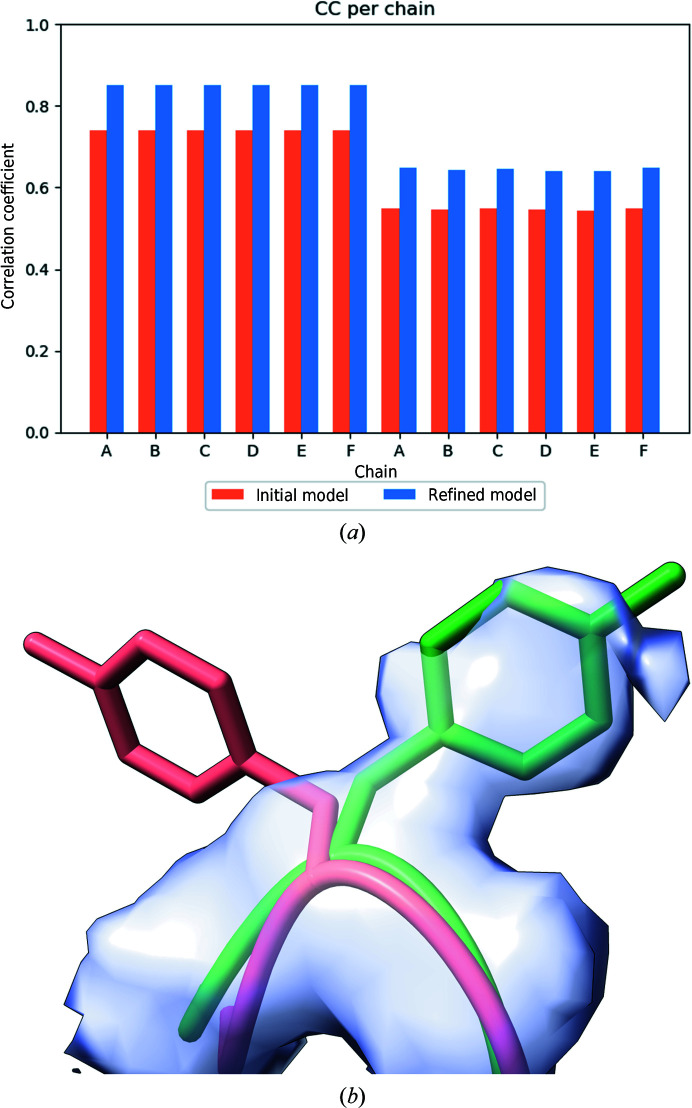
PDB entry 5k12. (*a*) CC per chain as shown on the website. The correlation coefficient per chain is divided into CC for protein residues and CC for ligands and water. (*b*) Residue Tyr471*F* before refinement (coral) and after refinement (green).

PDB entry 5k12 [8194] represents the cryo-EM structure of glutamate dehydrogenase obtained at 1.8 Å resolution (Merk *et al.*, 2016 ▸). The structure is composed of six protein chains, each with 294 residues (the full sequence length is 558) and some water molecules. All of the metrics in cryo-EM Table 1 improve after re-refinement (Table 1 ▸), such as the clashscore, *CaBLAM* outliers, Ramachandran metrics and CC_mask_. Most strikingly, the CC per residue improves systematically for most protein residues (Fig. 10 ▸ *a*). An example is residue Tyr471 (chain *F*; Fig. 10 ▸ *b*). In the initial model, the side chain points into a region without density, while in the re-refined structure the side chain flips to an area that is within density. This model was released in 2016 (the version of *Phenix* used at the time was not indicated). The fact that the current version of *phenix.real_space_refine* can improve this model further underscores the automated re-refinement and illustrates how the computational tools have improved over time. This model may have benefited from recent enhancements in side-chain fitting procedures, which improve the orientations of side chains and move them into density peaks. This is of particular interest for large models, where manual corrections are time-consuming.

#### 3.4.2. PDB entry 3j4p, 4.8 Å resolution   

PDB entry 6htx [5681] represents the cryo-EM structure of the adeno-associated virus obtained at 4.8 Å resolution (Xie *et al.*, 2013 ▸). The model is composed of 31 000 residues in 60 chains. While the model-to-map fit is essentially identical before and after re-refinement, all model-validation metrics improve significantly (Table 1 ▸). The model was released in 2013, when mature atomic refinement programs for cryo-EM models were not yet widely available. This highlights the benefit of revisiting older structures in the re-refinement project. We note that the model also contains 60 water molecules, 180 ions and sucrose octasulfate molecules (Na, Mg, SCR) and 120 alternative conformations. It is likely these features are remnants of the models that were used as templates for the virus structure. At 4.8 Å resolution, water molecules typically do not show up as clear density peaks. Ions may only be identified to some extent if they adopt their characteristic coordination.

## 4. The *CERES* website   

The website is hosted at https://cci.lbl.gov/ceres/. The content of the website is divided into different pages.

**Figure 11:**
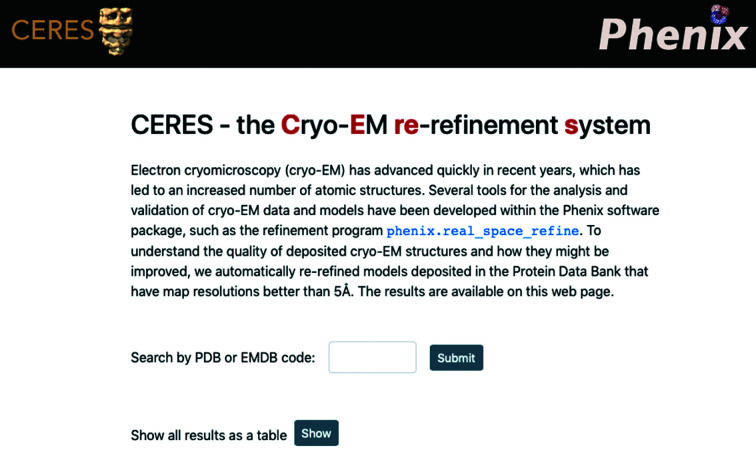
The *CERES* home page. The menu bar at the top allows navigation to the ‘About’, ‘Glossary’, ‘Figures PDB/EMDB’ and ‘Contact’ pages. The search field allows queries of the database with a PDB code or EMDB map code. The results for all structures can also be displayed as a table.

**Figure 12:**
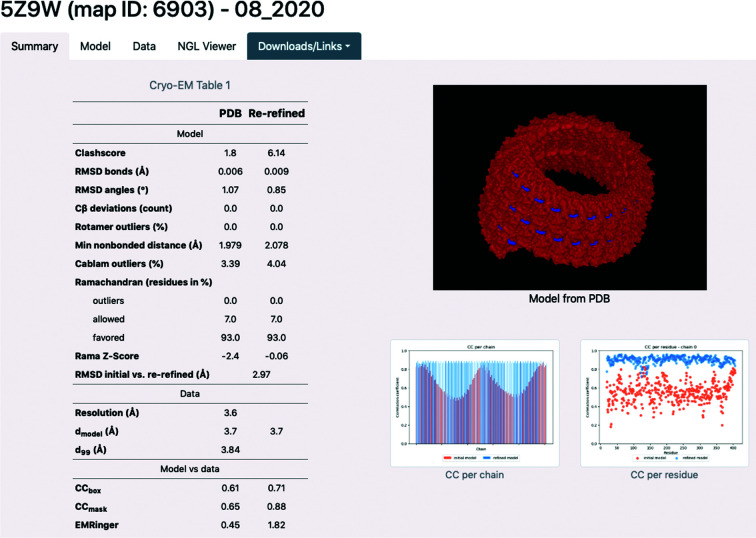
‘Summary’ tab on the individual results page. (*a*) The information is categorized in tabs. (*b*) Cryo-EM Table 1 ▸. (*c*) Embedding of the NGL viewer, showing the model as hosted on the PDB. (*d*) Plots showing the CC per chain and the CC per residue for each chain. When clicked, the images expand (CC per chain) or a gallery is opened (CC per residue).

**Figure 13:**
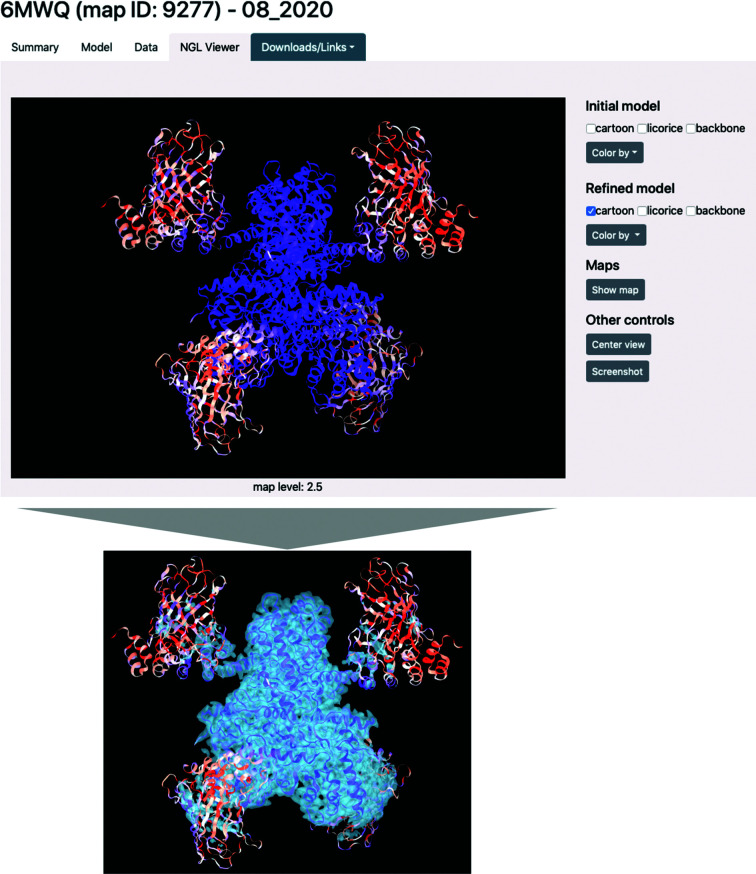
Embedding of the NGL viewer in the individual results page for PDB entry 6mwq [9277]. The viewport can display the initial and refined models and the map. The coloring is set according to CC per residue (blue, good fit; red, suboptimal fit). In this example, the outer parts of the molecule have lower CC values. The superposed map (bottom) has lower contour levels in these regions. The embedded viewer is therefore useful to quickly identify problematic regions with poor map-to-model fit.

*Home*. The starting page (Fig. 11 ▸) allows the database to be searched using a PDB or EMDB ID. Alternatively to searching for a particular entry, all results can be accessed in a sortable table via a button.
*About*. A description of the re-refinement procedure and an explanation of the tasks involved in the process.
*Glossary of terms*. Definition of the metrics shown for each entry.
*Figure PDB/EMDB*. Overview of cryo-EM maps and models. The page shows monthly updated figures for the fraction of major methods in the PDB (similar to Fig. 1 ▸), the number of cryo-EM models deposited in the PDB per year, the number of large deposited models (molecular weight of >1000 kDa) per year for X-ray and cryo-EM, and the resolution of deposited cryo-EM maps (similar to Fig. 2 ▸). The figures give a snapshot of the current state of cryo-EM depositions.
*Individual results page*. If an entry exists in the database for a given PDB or EMDB ID, a page summarizing re-refinement results and model properties is shown. An example for PDB entry 5z9w is displayed in Fig. 12 ▸. The individual results page is subdivided into four tabs. The ‘Summary’ tab shows a concise overview of the refinement results. It includes cryo-EM Table 1, with model, data and model-versus-data metrics as well as plots for map–model correlation coefficients. The NGL viewer is embedded, showing the model as hosted on the PDB. The ‘Model’ tab gives further information about the model composition (the number of chains, residues, atoms, ligands *etc.*) and model statistics, such as ADPs and occupancies. The tab also features Ramachandran plots and an atomic *B*-factor histogram. The ‘Data’ tab shows properties of the map file, such as box information (comparable to the unit cell in crystallography), several resolution estimates and map value statistics. A model-versus-map FSC curve is shown and if half-maps are available, their FSC plot is also displayed. The ‘NGL viewer’ tab (Fig. 13 ▸) contains a molecular visualization application. The initial and refined model files, as well as the boxed map, can be displayed.^5^ Via interface elements, the representation and coloring of the models can be changed and screenshots of the views can be downloaded. The viewer allows the rapid identification of problematic regions of the model, such as residues with low CC values or high *B* factors, via the coloring options. Furthermore, areas where the model is not covered by density can easily be seen. The results page also has links to the database entries of the original map and model in the EMDB and PDB, respectively. The log file from refinement can be opened and the refined model file can be downloaded.
*All results as a table*. The results from re-refinements can be also browsed in a responsive table. Each column is sortable, so that it is easy to find, for example, structures with the best map resolution or with the highest map–model CC. Columns can be hidden or added to optimize the table appearance according to the needs of the query.

## 5. Conclusion   

This work describes re-refinements of cryo-EM models at map resolutions of up to 5 Å with *phenix.real_space_refine*. 2535 model–map pairs were re-refined and the results are publicly accessible on a website. A significant number of models could not be re-refined because the automatic curation procedures were thwarted by inconsistent metadata information. Cryo-EM data, although dramatically better than before the recent innovations, are still often at low enough resolution to present challenges for model building, refinement and validation. Therefore, we were not surprised to observe that for those models that were successfully re-refined, the model-to-map fit and numerous geometric validation parameters generally improved. It is worth adding that a complete evaluation requires a robust measure of cross-validation, which is currently lacking. Therefore, the re-refinement results suggest that the current methods for constructing and refining models with cryo-EM data can be improved further, as can the best practices for practitioners of cryo-EM.

The results of the re-refinements also highlight some of the areas for improvement. As an example, the current algorithms in *Phenix* for Ramachandran restraints need to be better adapted to the features of low-resolution cryo-EM maps. Also, better validation metrics for assessing model-to-map agreement are required. The number of failures of automatic curation procedures argue for better standardization of metadata in map/model depositions, as well as the consistent collection of important data-validation information in the form of independent half-maps. The reanalysis of structures would be facilitated by the inclusion of more information in depositions. Half-maps could be potentially used as a measure for the resolution if the metadata give ambiguous results, but unfortunately the deposition of half-maps is not mandatory. To enable other researchers to judge the quality of cryo-EM structures, we suggest a number of validation metrics to be included in structural reports of cryo-EM models, similar to the ‘Table 1’ used in crystallography. To address the artifacts that we observed in some models, future improvements of the re-refinement server will include filtering of ligands and water molecules that do not have any signal in the map. Furthermore, we will incorporate validation metrics that are specific for nucleic acids.

## Figures and Tables

**Figure 3:**
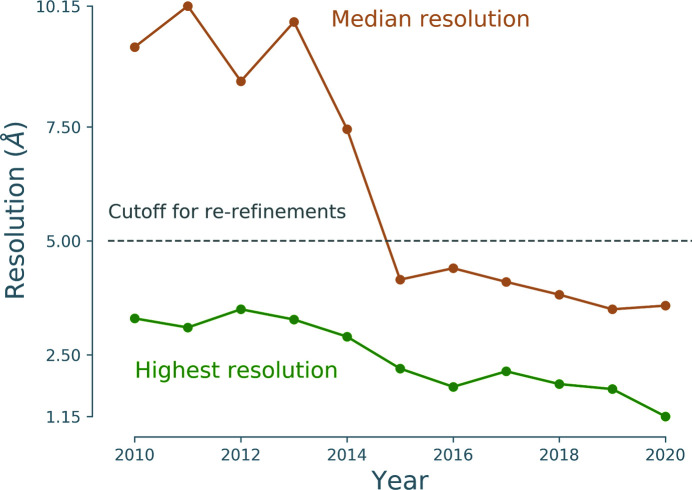
The median resolution of cryo-EM maps that have corresponding models in the PDB (orange) has improved steadily since 2013, achieving a median of better than 5 Å since 2015. The highest resolution also improved, with the best value being 1.15 Å for EMD-11668 (PDB entry 7a6a). Note that this figure refers to maps that have a corresponding model in the PDB.

**Table 1:** Model, data and model-versus-data metrics before and after refinement for two examples (PDB entries 5k12 and 3j4p)

	PDB entry 5k12 [8194]		PDB entry 3j4p [5681]	
	PDB	Re-refined	PDB	Re-refined
Model				
Clashscore	19.8	10.4	77.4	22.0
R.m.s.d.				
Bonds (Å)	0.009	0.007	0.012	0.004
Angles (°)	1.21	0.73	1.66	0.82
C^β^ deviations (count)	0.0	0.0	0.0	0.0
Rotamer outliers (%)	0.82	0.96	8.64	0.89
Minimum nonbonded distance (Å)	2.05	2.13	1.42	2.13
*CaBLAM* outliers (%)	0.7	0.64	7.41	2.42
Ramachandran plot (%)				
Outliers	0.0	0.0	4.3	0.0
Allowed	3.8	2.9	11.8	6.7
Favored	96.2	97.1	83.9	93.3
Rama-*Z* score	−2.53	1.79	−5.11	−2.48
R.m.s.d. initial versus re-refined (Å)	0.86		0.80	
Data				
*d*_FSC_ (Å)	—†		—†	
Consensus resolution (Å)	1.8		4.8	
*d*_model_ (Å)	2.8	2.8	5.0	5.0
*d*_99_ (Å)	2.41		5.13	
Model versus data				
CC_box_	0.66	0.70	0.9	0.91
CC_mask_	0.74	0.88	0.89	0.87
EMRinger	5.8	3.71	—‡	—‡

† *d*_FSC_ could not be calculated because half-maps were not available.

‡ The EMRinger score is calculated when the map resolution is better than 4 Å.

## Footnotes

1 However, phases can be inferred through experimental phasing approaches such as SAD, MAD, MIR *etc*.

2 Future versions will use the mmCIF format exclusively, as the PDB format is deprecated.

3 The numbers are approximate, as new entries are added to the EMDB weekly. Curation is once a month. Re-refinements are run once a month.

4 Here and subsequently, the number in square brackets denotes the EMDB code.

5 As the full map may be very large, leading to a frustrating loading time, the maps displayed in the viewer have been cut out around the model with *phenix.map_box*.
